# The Impact of a New “Inverted Arch” Prosthetic Annuloplasty Ring on the Mitral Valve’s 3-D Motion: An Experimental Ex-Vivo Study

**DOI:** 10.3390/bioengineering6020031

**Published:** 2019-04-08

**Authors:** Philippe Caimmi, Emmanouil Kapetanakis, Carla Beggino, Giovanni Vacca, Elena Grossini, Florian Stratica, Roberto Sacco, Andrea Capponi

**Affiliations:** 1 Department of Medical Direction of University Hospital of Novara, Corso Mazzini 18, 28100 Novara, Italy; florian.stratica@maggioreosp.novara.it (F.S.); roberto.sacco@maggioreosp.novara.it (R.S.); andre.capponi@maggioreosp.novara.it (A.C.); 2 Department of Cardiac Surgery, “Attikon” University Hospital, 12462 Athens, Greece; kapetanakis@hotmail.com; 3 Department of Cardiac Surgery, University Hospital of Novara, 28100 Novara, Italy; carlabeggino@yahoo.it; 4 Department of Experimental Surgery, University Hospital of Novara, 28100 Novara, Italy; giovanni.vacca@med.uniupo.it (G.V.); elena.grossini@med.uniupo.it (E.G.); 5 Department of Physiology, University of Eastern Piedmont “Amedeo Avogadro”, 28100 Novara, Italy

**Keywords:** mitral valve annuloplasty, mitral valve repair, prosthetic mitral ring

## Abstract

This experimental study aimed to evaluate the ex-vivo three-dimensional (3-D) motion of the Inverted Arch Ring (IAR), an innovative new design concept for a flexible incomplete annuloplasty prosthesis with an incorporated stabilizing rigid arch that can be used in correcting mitral valve regurgitation. Twenty explanted porcine hearts were placed in a circulation simulation system. Ultrasonometry transducers implanted in the mitral annulus were used to measure the 3-D valvular motion during a simulated cardiac cycle. Annular distance measurements were recorded and compared in each heart before and after the implantation of the IAR prosthesis at pressures corresponding to mid-systole and mid-diastole. Distances measured in mid-systole and mid-diastole demonstrated no significant differences in annular motion or in valve areas either prior to or after IAR implantation. Therefore, the results of this study confirm the minimal effects exerted by the IAR prosthesis on the mitral valve’s 3-D motion during a simulated cardiac cycle.

## 1. Introduction

Because of its important technical and clinical advantages, mitral valve (MV) annuloplasty has been established within the cardiac surgery scientific community as the “gold standard” repair approach for mitral valve disease [1,2,3]. However, despite its conservative nature and the preservation of anatomical structures, there is still a considerable trade-off in regards to distorting the native valve’s normal movement physiology, which affects the efficiency and durability of the repaired MV [4,5].

**Figure 1:**
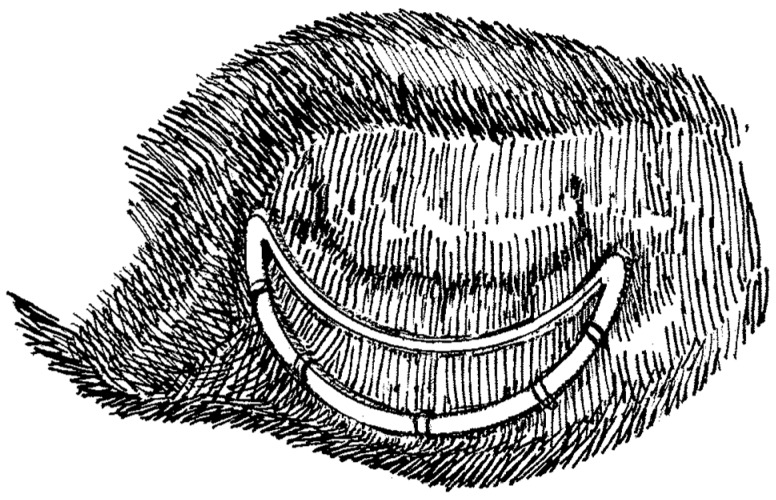
Illustration of the Inverted Arch Ring (IAR) prosthesis in situ as implanted following a mitral valve (MV) annuloplasty procedure.

The Inverted Arch Ring (IAR) is a new concept for a flexible incomplete prosthetic annuloplasty ring with a stabilizing rigid element that has been designed to correct MV dysfunction while minimizing the impact of the repair on annular motion. It is designed to be implanted like a regular annuloplasty prosthesis via the established mitral repair technique (Figure 1). The ex-vivo study presented herein was undertaken to evaluate and analyze the effects of the implantation of this new prosthesis on the three-dimensional (3-D) motion of the MV’s annulus during the cardiac cycle.

## 2. Materials and Methods

Twenty (20) explanted hearts from young female domestic pigs (*Sus scrofadomesticus)* of 55 kg average weight were utilized. Animals were weight-controlled so as to maintain a constant heart size and annular diameters to match our prototype ring prosthesis and to avoid an under or over-sizing annuloplasty. Harvested tissue was provided by the University’s animal husbandry facility, which complies fully with the European guidelines for the care of laboratory animals. All hearts were harvested after a single dose of Custodiol cardioplegia in anesthetized pigs that were used for surgical training courses of residents/trainee surgeons at our university and were subsequently scheduled to be euthanized. Furthermore, this study was performed ex-vivo. Therefore, ethical approval by our local ethical board was not required. Consent for tissue harvesting was obtained from our animal husbandry facility’s administration. These anatomical preparations were placed in a circulation simulation system and ex-vivo measurements were performed during a simulated complete cardiac cycle.

### 2.1. Description of the Prosthesis

**Figure 2:**
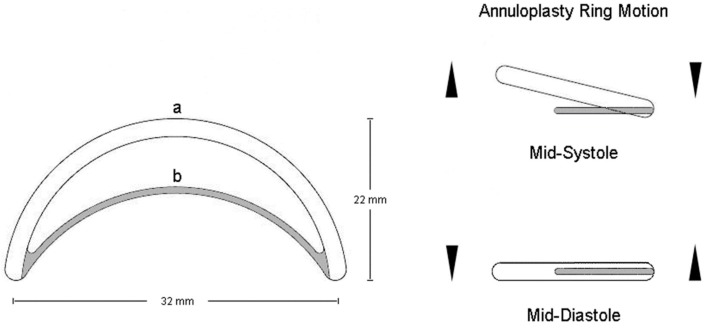
Inverted Arch Ring design with (**a**) a flexible semi-circumferential Polytetrafluoroethylene (PTFE) ring and (**b**) a stabilizing rigid inter-trigonal element.

The IAR was designed based on the principle of an inverted arch mechanism that is adapted to the posterior mitral leaflet’s shape and is composed by two elements: A) a completely flexible semi-circumferential Polytetrafluoroethylene (PTFE) ring that is designed to be sutured along the MV’s posterior annulus extending from the anterio-lateral to the posterior-medial trigone; and B) a rigid inter-triagonal arch of a metal alloy connecting the two triagones anteriorly (Figure 2).

The rigid inter-trigonal arch stays above the coaptation point and is never involved in leaflet motion. Furthermore, according to the concept of the “coaptation triangle”, the annuloplasty remodeling decreases the anterior-posterior annular diameter and increases the coaptation’s depth in order to improve the coaptation’s length. Therefore, the distance between the inter-trigonal rigid arch and the leaflets remains stable, avoiding any contact with the leaflets.

### 2.2. Circulation Simulation System Design

**Figure 3:**
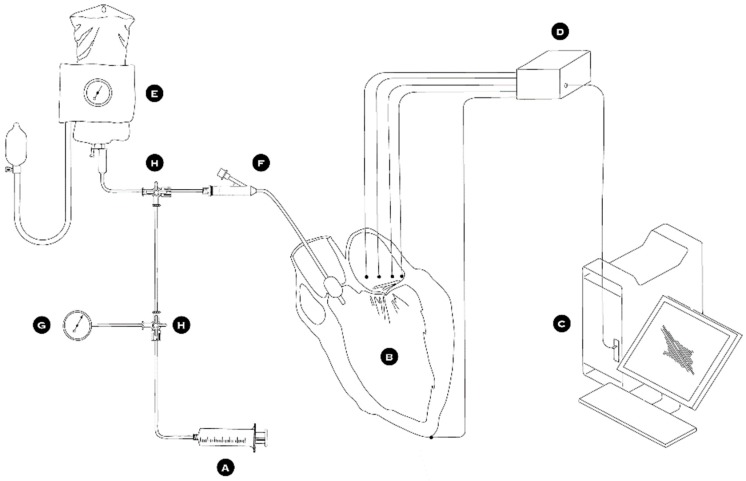
Schematic depiction of the circulation simulator system. (**A**) 60ml syringe with normal saline, (**B**) left ventricle, (**C**) computer with data acquisition and analysis program, (**D**) probes and signal acquisition module, (**E**) pressure cuff and normal saline bag, (**F**) foley catheter, (**G**) pressure manometer, and (**H**) three way stop-cock.

**Figure 4:**
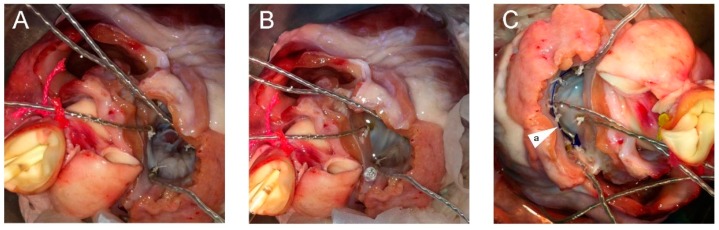
(**A**) Mitral valve orifice at mid-diastole, and (**B**) mid-systole. (**C**) Implantation of the (a) Inverted Arch Ring on the posterior mitral annulus.

**Figure 5:**
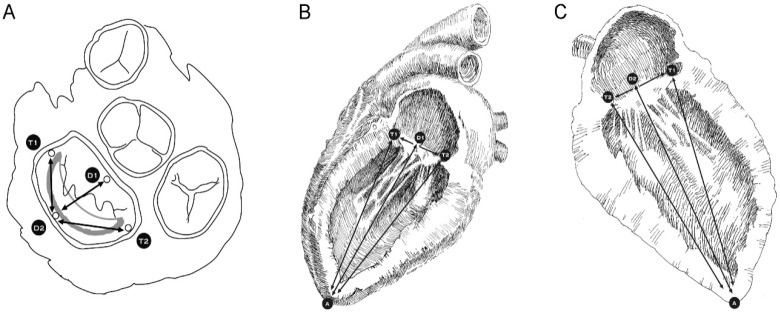
Anatomical positions, (**T1**, **T2**, **D1**, **D2**, and **A**) in the transverse and sagittal planes of the ultrasonometry measuring transducers and distances measured between ultrasonometry transducers in the (**A**) transverse, (**B**) anterior sagittal, and (**C**) posterior sagittal planes.

The complete setup of the circulation simulation system is depicted in Figure 3. First, the ascending aorta was snagged via a silk tie while the pulmonary veins were ligated with heavy silk suturing. A deflated 16 Fr Foley catheter and a pressure measuring probe were introduced into the ascending aorta through the aortic valve. This pressure-measuring catheter was used to take measurements during all phases of the experiment and control the simulated intra-ventricular pressure. The silk tie snugger was tightened around the ascending aorta causing the left ventricle to become watertight and so be able to be pressurized. A special collar was used around the two catheters to protect them from constriction. To simulate the cardiac cycle, an inflation/deflation pressurization system was used to pump normal saline into and out of the left ventricle (Figure 3A,E,F,G,H). The MV was exposed through the roof of a left atriotomy (Figure 4). Five hemispherical 2-mm size piezoelectric ultrasonometry transducers (model PZT-5A, Sonometrics Corp., London, Ontario, Canada) were implanted in the mitral ring, one on the anterior-lateral trigone (Figure 5, T1), one on the posterior-medial trigone (Figure 5, T2), one on the median axis of the anterior portion of the mitral ring (Figure 5, D1), one on the median axis of the posterior portion of the mitral ring (Figure 5, D2), and finally one at the top of the left ventricle (Figure 5A). These ultrasonometry transducers were held in position via pre-placed silk sutures on the native MV annulus and their cable wires were passed free through the left atriotomy (Figure 4). Baseline-control measurements were taken, and subsequently the prototype IAR prosthesis was implanted into the MV’s posterior annulus (Figure 4C) and all measurements were repeated.

### 2.3. Data Acquisition

During the experiment, data were generated in analog format by piezoelectric crystals and transmitted via the cable wires to an acquisition module where the 800 kHz pulses were processed and converted into digital signals. From there, the signals were transferred to a personal computer (PC) station where measurements were actualized and recorded by the processing software (Sonometrics Corp., London, Ontario, Canada).

Two sets of measurements were recorded: one at a minimal pressure of 5 mmHg corresponding to ventricular mid-diastole (Figure 4A) and one at the maximal pressure of 120 mmHg, which corresponds to mid-systole (Figure 4B).

A number of measurements were generated, which included the anterio-posterior annular distance (between D1 and D2) (Figure 5A), the inter-trigonal diameter (between T1 and T2) (Figure 5B), the anterior semi-trigonal distance (between D2 and T1) (Figure 5A), and the posterior semi-trigonal distance (between D2 and T2) (Figure 5A). In addition, the anterio-lateral trigone to the apex distance (between T1 and A) (Figure 5B), the posterio-medial trigone to the apex distance (between T2 and A) (Figure 5B), the anterior annulus to apex distance (between D1 and A) (Figure 5B), and the posterior annulus to apex distance (between D2 and A) (Figure 5C) were measured. The annulus to apical distances (T1–A, T2–A, D1–A, D2–A) are indicative of the prosthesis’ movement above and below the MV’s annular plane, while the semi-trigonal distances (D2–T1, D2–T2) represent the parameter indicative of the deformation in the circumferential shape of the flexible portion of the prosthesis. Finally, the anterio-posterior distance (D1–D2) combined with the inter-triagonal diameter (T1–T2) allows for the calculation of the effective valve area, assuming that it has the area of an ellipse. All these measurements were recorded both in mid-systole and mid-diastole before and after the implantation of the IAR in each heart and under the controlled pressurization parameters presented above.

### 2.4. Statistical Analysis

All data are reported as mean value ± standard deviation. The averages of measurements before and after IAR implantation were compared, with each heart acting as its own control group. The paired Student’s t-test assuming normal distributions was used for the analysis. A *p*-value of 0.05 or less was considered to be statistically significant. All statistical analyses were performed using the statistical software program Stateview 5.0 (StataCorp LP, College Station, TX, USA).

## 3. Results

Both baseline/control measured distances prior and after IAR implantation demonstrated the expected statistically significant differences in mid-systole and mid-diastole, which correspond to changes due to the physiologic dynamic movement pattern of the MV that has been reported in the literature [6,7,8]. Specifically, the effective orifice area at baseline prior to prosthesis implantation changed from 5.89 ± 1.1 cm^2^ in mid-systole to 6.26 ± 1.7 cm^2^ in mid-diastole (*p* = 0.035), which is equivalent to an increase of 9.4%, while, following IAR implantation, the MV’s effective area changed from 5.69 ± 0.8 cm^2^ in mid-systole to 6.08 ± 1.2 cm^2^ in mid-diastole (*p* = 0.039), which corresponds to an increase of 9.35% (Table 1).

Similarly, in regard to potential valvular deformation following the performance of an annuloplasty, the semi-trigonal distances again demonstrated a predictable symmetrical behavior during the cardiac cycle before and after IAR implantation; the D2–T1 distance in mid-systole was 17.8 ± 1.2 mm and 15.9 ± 1.3 mm in mid-diastole (*p* = 0.028), while the D2–T2 distance in mid-systole was 17.5 ± 0.2 mm and 15.8 ± 0.8 mm in mid-diastole (*p* = 0.022) (Table 1).

Finally, when evaluating the annular movement on the vertical axis, the apical distances prior to and following prosthesis implantation again showed the statistically significant changes attributable to the physiologic movements the MV exhibits during the cardiac cycle. Specifically, prior to IAR implantation the T1–A distance was 89.8 ± 13 mm in mid-systole and 103.6 ± 6 mm in mid-diastole (*p* = 0.026), the T2–A distance was 90.1 ± 18 mm in mid-systole and 109.4 ± 13 mm in mid-diastole (*p* = 0.024 ), the D1–A distance was 90.4 ± 14 mm in mid-systole and 107.7 ± 10 mm in mid-diastole (*p* = 0.028), and the D2–A distance was 92.6 ± 16 mm in mid-systole and 112.3 ± 12 mm in mid-diastole (*p* = 0.027) (Table 1). Subsequent to IAR implantation, the vertical movement pattern of the annulus continued to be unaffected with the T1–A distance being 90.5 ± 11 mm in mid-systole and 106.6 ± 12 mm in mid-diastole (*p* = 0.025), the T2–A distance being 90.2 ± 19 mm in mid-systole and 108.6 ± 12 in mid-diastole (*p* = 0.027), the D1–A distance being 93.1 ± 17 mm in mid-systole and 111.6 ± 14 mm in mid-diastole (*p* = 0.025 ), and the D2–A distance being 91.9 ± 18 mm in mid-systole and 109.8 ± 17 mm in mid-diastole (*p* = 0.032) (Table 1).

A further comparison performed to investigate possible differences on the annular anatomy and the valve’s 3-D motion pattern during the cardiac cycle that may have been caused by the implantation of the IAR prosthesis demonstrated no significant differences in distance values or valve areas prior to and after annuloplasty either in mid-systole or in mid-diastole.

## 4. Discussion

The MV is located at the base of the left ventricle, anchored to the fibrous trigones, and is separated and divided from the aortic valve by the aorto-mitral membrane [5]. The upper portion of this membrane comprises part of the aortic valve’s annulus while the lower portion continues as the MV’s anterior leaflet [5]. The MV’s annulus undergoes significant 3-D transformations in its morphology during the cardiac cycle, modifying its effective orifice area. According to the literature, these changes in the effective orifice area can range from 6.9% to 16.7% in an ovine model and from 9.3% to 26% in healthy human volunteers [6]. The authors have previously demonstrated that repairing the MV with a rigid complete prosthetic ring immobilizes the aorto-mitral membrane and thus impairs the filling and emptying mechanism of the left ventricle [5]. In fact, rigid complete prosthetic rings tend to fix the MV’s annulus in a set systolic orientation abolishing the physiologic aorto-mitral and hinge-saddle movements of the valve.

Furthermore, this fixation of the MV’s annular movements increases significantly the stress on ring sutures and on the mitral chords increasing the risk for ring dehiscence and mitral chord failure/rupture [9,10,11]. Although the rigid incomplete ring designs provide no fixation of the aorto-mitral membrane, they too interfere with the hinge-saddle motion of the posterior portion of the MV annulus. In contrast, semi-rigid or entirely flexible incomplete annuloplasty rings are not able to effectively stabilize the geometry of the MV annulus, thus rendering the repair ineffective and not durable. Recognizing these limitations has prompted the authors to develop an entirely flexible incomplete annular prosthesis with an ancillary stabilizing rigid arch in order to preserve the flexibility of the Aorto-Mitral membrane and the 3-D motion of the entire MV annulus while concurrently providing improved stabilization.

The prototype of this new prosthesis has been constructed at the laboratory of experimental surgery of our university and is of an incomplete annular design that is composed posteriorly by a flexible semi-circumferential strip of PTFE that is closed anteriorly by a rigid inter-trigonal elliptical arch of a metal alloy (Figure 2). The flexible strip is sutured on the posterior portion of the annulus with the elliptical rigid arch spanning each trigone and laying across the valve’s orifice, just above the free margin of the MV’s anterior leaflet, consequently avoiding leaflet interference and any associated turbulent blood flow (Figure 1 and Figure 4C). This design allows for the motion and displacement of the flexible annular ring along with the MV’s normal 3-D movement while concurrently securing at mean time in space the anterio-lateral and posterio-medial trigones and the posterior portion of the annulus, thus correcting the annular shape and preventing further dilatation. Furthermore, the absence of prosthetic structures and sutures on the anterior portion of the MV’s annulus allows for the preservation of the physiological motion of the aorto-mitral membrane.

In this experimental work, we studied the impact of the IAR prosthesis on the 3-D functionality of the Mitral annulus. So as to ensure absolute reproducibility, the experiment was performed ex-vivo on a series of isolated healthy porcine hearts placed on a circulation simulation system. Each heart acted as its own control with systole and diastole being simulated under monitored, identical hemodynamic conditions prior to and after the implantation of the prosthetic ring. This ex-vivo approach allows for the elimination of functional bias caused by the instigation and cessation of cardiopulmonary bypass.

The healthy porcine model, utilized naturally, does not reproduce pathological annular dilatation, and this could be construed as a limitation to the study. However, dilated mitral annuli usually have an impaired 3-D movement. Because this was a proof of concept study and the endpoint of the experiment was to measure the impact of the IAR prosthesis on the mitral valve’s 3-D motion, a healthy cardiac model was utilized that was much easier to procure, more reproducible, and, we feel, more applicable because its 3-D motion is not impaired by annular dilatation.

The use of multi-planar ultrasonometry allowed us to precisely monitor the motion of the IAR in all planes and positions in space during the cardiac cycle. We were able to demonstrate that the implantation of the IAR did not affect the changes in mid-systolic and mid-diastolic distances or in effective orifice areas compared to baseline significantly. In particular, we observed the preservation of the physiological mid-diastolic increase in anterio-posterior diameter (D1–D2) and in valvular area following IAR implantation (9.4% versus 9.35%). The characteristic mid-systolic to mid-diastolic change in the anterior annulus to apical distance (D1–A) remained unaffected before and after IAR implantation. This, in correlation with the preservation of the change in anterio-posterior diameter (D1–D2) mentioned above, indirectly confirms that IAR implantation does not alter the anterior-posterior motion of the aorto-mitral membrane. This is attributed to the fact that the ring prosthesis is implanted only in the posterior portion of the annulus. Similarly, the preservation of the changes in the posterior annulus to apical distances (T1–A, T2–A, and D2–A) demonstrate the preservation of the 3-D hinge-saddle movement during the cardiac cycle. This is attributed to the flexibility of the PTFE strip implanted on the posterior annulus. Finally, the prosthetic ring maintained its perfectly symmetrical shape despite its intrinsic flexibility even under maximum pressure loads and traction. This finding is demonstrated by the stability in the semi-trigonal distances (D2–T1 and D2–T2) exhibited during the cardiac cycle following IAR implantation. This is attributed to the support provided by the ancillary rigid metal arch.

Through this study, we have tried to present an inventive new design concept for an MV annuloplasty prosthesis. Our observations substantiate the stabilization properties of the IAR prosthesis and the concomitant absence of detrimental effects on 3-D annular mobility.

Although this device is still in the prototype phase and obviously requires further refinement and significant additional ex-vivo and in-vivo testing, especially in beating heart experiments to confirm the resilience and physiological benefits of the design, we hope that the presentation of this new ring concept and our initial results to the cardiac surgery and bioengineering communities will prompt further discussion and refinement and stimulate further research and innovative similar designs.

## 5. Conclusions

The findings of this ex-vivo study have shown the advantageous structural and dynamic movement properties of the new IAR annular prosthesis we sought to demonstrate during the initial design phase. The IAR is an innovative and original design concept for mitral valve remodeling and stabilization that appears to cause minimal or no impact on the valve’s 3-D motion. These findings are therefore supportive for continuing the development and assessment of the IAR prosthesis utilizing a more complex beating/live heart model.

## Figures and Tables

**Table 1:** Comparison of Distance Measurements during the Cardiac Cycle prior to and following an Inverted Arch Ring Annuloplasty.

Distance Measurements	Mid-Systole		Mid-Diastole		*p*-Value ^1^	*p*-Value ^2^
	Without IAR Prosthesis	With IAR Prosthesis	Without IAR Prosthesis	With IAR Prosthesis		
**D1–D2** (mm)	23.3 ± 4	22.8 ± 2	24.7 ± 2	24.3 ± 4	0.037	0.031
**T1–T2** (mm)	32.1 ± 5	31.8 ± 2	32.3 ± 4	31.9 ± 2	0.5	0.54
**D2–T1** (mm)	18.3 ± 0.6	17.8 ± 1.2	16.7 ± 0.9	15.9 ± 1.3	0.025	0.028
**D2–T2** (mm)	17.9 ± 0.9	17.5 ± 0.2	16.1 ± 1.0	15.8 ± 0.8	0.035	0.022
**T1–A** (mm)	89.8 ± 13	90.5 ± 11	103.6 ± 16	106.6 ± 12	0.026	0.025
**T2–A** (mm)	90.1 ± 18	90.2 ± 19	109.4 ± 13	108.6 ± 12	0.024	0.027
**D1–A** (mm)	90.4 ± 14	93.1 ± 17	107.7 ± 10	111.6 ± 14	0.028	0.025
**D2–A** (mm)	92.6 ± 16	91.9 ± 18	112.3 ± 12	109.8 ± 17	0.027	0.032
**Valve Area** (cm^2^)	5.89 ± 1.1	5.69 ± 0.8	6.26 ± 1.7	6.08 ± 1.2	0.035	0.039

^1^ *p*-value of comparison of measurements in mid-systole and mid-diastole prior to IAR annuloplasty.^2^ *p*-value of comparison of measurements in mid-systole and mid-diastole following IAR annuloplasty.
